# Amyloidosis cutis dyschromica in two female siblings: cases report

**DOI:** 10.1186/1471-5945-11-4

**Published:** 2011-02-15

**Authors:** Wenlin Yang, Yangyang Lin, Jian Yang, Wensheng Lin

**Affiliations:** 1 Department of Dermatology, the Second Affiliated Hospital of Guangzhou Medical University, Guangzhou 510260, China

## Abstract

**Background:**

Cutaneous amyloidosis has been classified into primary cutaneous amyloidosis (PCA, OMIM #105250), secondary cutaneous amyloidosis and systemic cutaneous amyloidosis. PCA is the deposition of amyloid in previously apparent normal skin without systemic involvement. Amyloidosis cutis dyschromica (ACD) is a rare distinct type of PCA. Here, the unique clinical and histological findings of two Chinese female siblings with ACD were described.

**Cases presentations:**

Patient 1 was a 34-year-old female, presented with mildly pruritic, diffuse mottled hyperpigmentation and hypopigmentation. The lesions involved all over the body since she was 10 years old. There were a few itchy blisters appearing on her arms, lower legs and truck, especially on the sun-exposed areas in summer. Some hypopigmented macules presented with slight atrophy. Patient 2 was 39-year-old, the elder sister of patient 1. She had similar skin lesions since the same age as the former. The atrophy and blisters on the skin of the patient with amyloidosis cutis dyschromica have not been described in previous literature. Histological examinations of the skin biopsies taken from both patients revealed amyloid deposits in the whole papillary dermis. Depending on the histological assessment, the two cases were diagnosed as amyloidosis cutis dyschromica.

**Conclusion:**

The two cases suggest that the atrophy and blisters may be the uncommon manifestations of amyloidosis cutis dyschromica. It alerts clinicians to consider the possibility of ACD when meeting patients with cutaneous dyschromia. Skin biopsy is essential and family consultation of genetic investigation is very important in such cases.

## Background

Cutaneous amyloidosis has been classified into primary cutaneous amyloidosis (PCA, OMIM #105250), secondary cutaneous amyloidosis and systemic cutaneous amyloidosis. PCA is the deposition of amyloid in previously apparent normal skin with no systemic involvement. Amyloidosis cutis dyschromica (ACD) is a rare distinct type of PCA. Here, the unique clinical and histological findings of two Chinese female siblings with ACD were described.

## Cases presentations

Patient 1 was a 34-year-old female who presented with mildly pruritic, diffuse mottled hyperpigmentation and hypopigmentation. The lesions involved all over the body since she was 10 years old. The hypopigmentated macules were first noticed on her bilateral lower legs, and had slowly progressed to involve the trunk, the upper limbs, the thigh, the neck and the face with age growing. She also noticed reticular hyperpigmentation macules surrounding the spotty hypopigmentation. There were a few itchy blisters which were sesame-like or mung-bean-like in size appearing on her arms, lower legs and truck, especially on the sun-exposed areas in summer and the blisters were easy to burst. She did not have the history of extensive sun exposure or any other systemic or cutaneous disease before the onset of the lesions.

**Figure 1:**
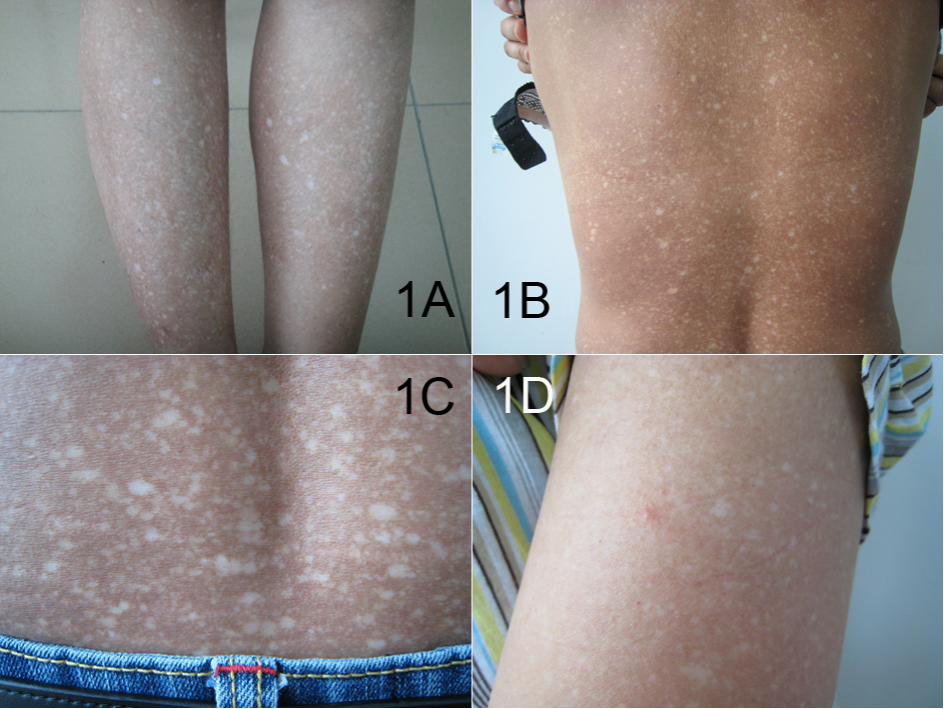
**The clinical features of the patient 1 with ACD**. The hyperpigmented and hypopigmented macules on (A) lower legs, (B) back and waist, (C) waist. (D) Individual blisters on upper arm.

On physical examination, she was well nourished and her stature was 158 cm. The cutaneous examinations revealed generalized, mottled, varying-sized hyperpigmented and hypopigmented macules ranging from 4 to 18 mm involving almost the entire body in a symmetrical pattern. In addition, some hypopigmented macules presented with slight atrophy. The skin lesions were more pronounced on the lower legs (Figure 1A), back and waist (Figure 1B, 1C), and the larger hypopigmented lesions were seen mainly on the lower legs. Individual blisters which were negative Nikolsky sign occurred on the upper arm (Figure 1D). The neck and face were only mildly affected. There was no apparent papular, erythema and telangiectasia. The dorsa of both hands and feet were uninvolved. The hair, teeth, nails, the mucosa of the mouth, palms, soles and vulva were normal-looking. The hearing was normal. Systemic examinations were unremarkable. The mental and developmental milestones of the patients were normal.

Patient 2 was 39-year-old, the elder sister of patient 1. Her stature was 149 cm. She had similar skin lesions since the same age as patient 1. She presented with diffuse hyperpigmentation and spotted hypopigmentation especially on the lower legs and the waist. She had less skin lesions than Patient 1, and did not show any pigmentation on the face or neck. Likewise, her systemic examination was unremarkable.

They were born to nonconsanguineous parents. Their mother had a small quantity of asymptomatic hypopigmented spots ranging from 2 to 4 mm in diameter on bilateral lower legs and back which were not explored further. Other family members have not similar manifestation.

**Figure 2:**
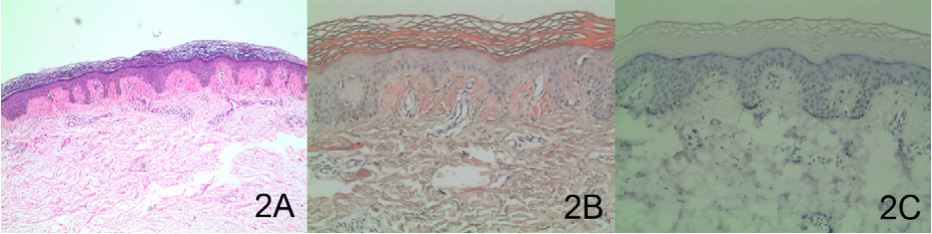
**The histological and immunohistochemical examinations of the patient with ACD**. (A) H&E staining indicated hyperkeratotic epidermis and amorphous eosinophilic masses in the papillary dermis. (original magnification × 40). (B) Congo red staining indicated positive for eosinophilic masses. (original magnification × 100). (C) The immunohistochemical examination indicated negative for HMB-45. (original magnification × 100)

The results of skin biopsies taken from the lower legs in both patients were similar. The pathological section with haematoxylin and eosin stain revealed hyperkeratotic epidermis and papillary dermis fully filled with amorphous eosinophilic masses (Figure 2A). The Congo red staining was positive to eosinophilic masses (Figure 2B), which indicated a deposit of amyloid substance. If using polarized light microscope, eosinophilic masses stained with Congo red will show the characteristic of apple-green birefringence. Additional staining with a marker of HMB-45, in order to differentiate ACD from other diseases with the characteristics of cutaneous dyschromia, was negative (Figure 2C).

Other results of routine laboratory examinations including routine blood test, biochemical profile, urine protein excretion and chest X-ray, abdominal ultrasound were normal in both patients.

Based on the clinical and pathological findings, the diagnosis of amyloidosis cutis dyschromica (ACD) was made for both patients. Topical tazarotene was prescribed without obvious improvement over a 1-month follow-up period.

## Discussion

PCA includes the more common papular (lichen amyloidosis) and macular types and nodular or tumefactive form [1]. ACD is a more uncommon subtype of PCA and was initially described by Morishima in 1970. This disorder is characterized by the following features: (i) dotted, reticular hyperpigmentation with hypopigmented spots without papulation almost all over the body; (ii) no or little itchy sensation; (iii) onset before puberty; and (iv) small foci of amyloid closely under the epidermis [2,3]. Eng et al [4] first reported family survey with ACD. Huang et al [5] and Choonhakarn et al [6] collected four cases from two families and six cases from three families respectively. Vijaikumar et al [7] reported two siblings with ACD.

Our cases presented with reticular hyperpigmentation with hypopigmented spots, which were generalized distribution and onset before puberty. The amyloid deposits were limited to the subepidermal region, without other cutaneous and systemic involvement. All of these were ranged ACD. Besides, in our patients, there are some clinical features, dotty atrophy and blisters of skin, that have not been described in previous literature of ACD.

Besides ACD, there are many other diseases with the characteristics of cutaneous dyschromia, including dyschromatosis universalis hereditaria, xeroderma pigmentosum and poikloderma-like amyloidosis. It is necessary to differentiate these diseases [3,8-10]. Amyloid deposits can not be founded in the skin of the patients with dyschromatosis universalis hereditaria and xeroderma pigmentosum,. ACD and poikyloderma-like cutaneous amyloidosis have similar clinical features. Poikyloderma-like cutaneous amyloidosis is characterised by the presence of poikylodermic lesions, lichenoid papules and blisters especially located on the limbs and appears in adult life. It is associated with photosensitivity, low height and a certain degree of palmo-plantar keratoderma. A few clinical features are overlapping between ACD and poikloderma-like amyloidosis. In our patients, there were dotty atrophy and vesicles of skin but no telangiectasia and palmo-plantar keratoderma. Also their statures were normal. Hence we do not know whether the two type of PCA belong to the same kind of disease, or there is a new subtype between the two types?

The etiology of PCA remains unknown, but it is believed to be multifactorial. Our patients are two female siblings and their mother has suspicious ACD. Owing to the familial aggregation, it suggests that genetic factors may play an important role in its pathogenesis. Vijaikumar et al [7] proposed that genetic factors may lead to UVB sensitivity and DNA repair defects. This repeated damage to the keratinocytes results in the production of amyloid materials in the skin [1]. However, genetic loci for familial PCA (FPCA) have not been identified so far. The clinical features of patients with PCA are varied. Whether genetic variability determines the forms of expression of the disease remains unknown.

Although the aetiology of PCA is not clear, some researches still help to explore the skin disorder whether linked to genetic pathogenesis. Lin's team [11] implied that a possible susceptibility locus for a subset of FPCA might exist on chromosome 1q23. By further experiments, an evidence of significant linkage to chromosome 5p13.1-q11.2 in a subset of FPCA was shown [12]. Lee et al [13] elucidated that evidence was lack to support for linkage of FPCA with the pericentromeric region of chromosome 10. Arita et al [14] indicated missense mutations of familial primary localized cutaneous amyloidosis (FPLCA) were located in the oncostatin M-specific receptor (OSMR) gene, which encodes oncostatin M-specific receptor beta (OSMR*β*) --a component of the oncostatin M (OSM) type II receptor and the interleukin (IL)-31 receptor. Their OSMR data in individuals with FPLCA represented the first human germline mutations in this cytokine receptor complex. PCA can be caused by mutations in two biologically associated cytokine receptor genes located on chromosome 5 [15]. Novel IL31RA gene mutation and ancestral OSMR mutant allele provide an explanation for the genetic pathogenesis of PCA. Tanaka's study [16] demonstrated that most changes of gene expression revealed alterations in epidermal differentiation and proliferation consistent with lichenification. They also found a reduction of several interfollicular keratinocyte stem cell markers in FPLCA skin. Their results promoted the molecular skin pathological progression on FPLCA.

The treating method of PCA is poor. topical corticosteroids, tretinoin, cryotherapy and carbon dioxide laser have varying effects. Systemic acitretin have been applied and suggest a good response [17].

## Conclusion

Our cases suggest that the atrophy and blisters may be the uncommon manifestations of amyloidosis cutis dyschromica. It alerts clinicians to consider the possibility of ACD when meeting patients with cutaneous dyschromia. Skin biopsy is essential and family consultation of genetic investigation is very important in such cases.

## Consent

Written informed consent was obtained from the patients for publication of this case report and any accompanying images. A copy of the written consent is available for review by the Editor-in-Chief of this journal

## Abbreviations

ACD: Amyloidosis cutis dyschromica; PCA: primary cutaneous amyloidosis; FPLCA: familial primary localized cutaneous amyloidosis; HE: haematoxylin and eosin. HMB-45: human melanoma black 45; OSM: oncostatin M; OSMR: oncostatin M-specific receptor; OSMR*β*: oncostatin M-specific receptor beta.

## Competing interests

The authors declare that they have no competing interests.

## Authors' contributions

WLY drafted the manuscript and designed the case-study. YYL participated in the data collection, followed up and managed the patient. JY carried out the histological examination, diagnosed and investigated. WSL participated in the data collection. All authors have read and approved the final manuscript.

## Pre-publication history

The pre-publication history for this paper can be accessed here:

http://www.biomedcentral.com/1471-5945/11/4/prepub
